# Effect of Artificial Aging Treatment on the Mechanical Properties and Regulation of Precipitated Phase Particles of High-Pressure Die-Cast Thin-Wall AlSi10MnMg Longitudinal Carrier

**DOI:** 10.3390/ma16124369

**Published:** 2023-06-14

**Authors:** Xu Zhao, Ping Wang, Yang Yang, Song Wang, Qiang Zhao, Jingying Sun

**Affiliations:** 1 Key Laboratory of Electromagnetic Processing of Materials, Ministry of Education, Northeastern University, Shenyang 110819, China; 2 BMW-Brilliance Automotive Ltd., Shenyang 100044, China; 3 Shenyang Wesszin Technology Co., Ltd., Shenyang 110044, China

**Keywords:** single-stage aging, double-stage aging, AlSi10MnMg alloy, microstructure, mechanical properties

## Abstract

This study investigates the artificial aging treatment process for AlSi10MnMg longitudinal carriers with optimal strength and ductility. Experimental results illustrate that the peak strength is observed under single-stage aging at 180 °C × 3 h, with a tensile strength of 332.5 MPa, Brinell hardness of 133.0 HB, and elongation of 5.56%. As aging time increases, tensile strength and hardness initially increase and then decrease, while elongation displays an inverse pattern. The amount of secondary phase particles at grain boundaries increases with aging temperature and holding time, but stabilizes as aging progresses; the secondary phase particles begin to grow, eventually weakening the alloy’s strengthening effect. The fracture surface exhibits mixed fracture characteristics, including ductile dimples and brittle cleavage steps. Range analysis indicates that the influence of distinct parameters on mechanical properties post-double-stage aging is as follows: first-stage aging time, first-stage aging temperature, followed again by second-stage aging time, and second-stage aging temperature. For peak strength, the optimal double-stage aging process includes a first-stage aging temperature of 100 °C × 3 h and a second-stage aging temperature of 180 °C × 3 h.

## 1. Introduction

In recent years, burgeoning sales of electric vehicles have catalyzed a trend towards lightweight construction in the automotive industry [1,2]. DuckerWorldwide published that the average aluminum content of European-produced cars is to reach nearly 180 kg in 2019, and the number will increase to almost 200 kg per vehicle by 2025 [3,4]. Studies show that a 10% weight reduction in an electric vehicle’s aluminum alloy body leads to a 5.5% decrease in energy consumption and a corresponding 5.5% increase in driving range. Notably, the additional battery costs required to achieve an equivalent range are significantly higher [5,6]. Die-cast aluminum alloys are more prevalent in automotive manufacturing than wrought aluminum alloys, making the development of aluminum alloy die-casting technology and its associated heat treatment processes a crucial area of research [7,8,9,10]. High-quality AlSi10MnMg aluminum alloy castings are extensively used in manufacturing automotive structural components, such as housing towers, longitudinal carriers, and transverse bearing beams [11,12,13,14].

Age-hardening, a heat treatment process, enhances an alloy’s strength by exploiting precipitation or dissolution behavior within an oversaturated solid solution obtained after solution treatment [15,16]. Artificial age-hardening techniques substantially enhance the strength of aluminum alloys after the solution treatment, with the T6 aging process manifesting the maximum mechanical strength of the alloy [17,18,19]. Lumley et al. reported significant fortification of A360 to die castings, even when the solution treatment phase was executed at 450 °C for merely 15 min [20]. Furthermore, Srivastava et al. postulated that a solution treatment at 480 °C for only 4 min could achieve compositional homogeneity in an Al–9%Si–Mg–Mn die-casting alloy, thereby increasing the yield strength to 200 Mpa [21]. 

Researchers have pioneered numerous age-hardening processes, including preaging, nonisothermal preaging, load compression aging, and double-stage aging [22,23]. Double-stage aging intensifies the alloy’s strength, hardness, and fracture toughness, thus establishing it as a superior method for augmenting the overall performance of aluminum alloys [24,25]. Favorable outcomes can be derived through graded aging or multistage aging heat treatments [26,27]. Ohishi et al. performed a study on the transformation mechanisms of precipitated phases in Mg–Zn–Al alloys during multistage aging [28]. Notably, double-aging resulted in significant alterations in the morphology and distribution of the precipitates, in contrast to single-aging [29]. Graded aging prompts the nucleation of the alloy at the preaging temperature of the first stage, subsequently followed by a gradual growth at ensuing aging temperatures [30]. This strategy facilitates the alloy in achieving enhanced mechanical properties within a relatively shorter duration, thereby augmenting the efficiency of the heat treatment process. When juxtaposed with conventional single-aging treatments, double-aging considerably bolsters the alloy’s tensile strength, elongation, and hardness [31].

Employing lightweight aluminum alloys as substitutes for steel in automotive body structural components must not entail any compromise on vehicle performance [32]. Even though the die-cast AlSi10MnMg alloy demonstrates significant tensile strength and hardness properties after T6 solution aging, further amplification of these characteristics, along with elongation, is crucial. Existing research provides limited insight into the mechanical properties and precipitate phases of AlSi10MnMg alloy after double-aging; consequently, this study aims to investigate the double-stage aging mechanism of AlSi10MnMg alloy, predicated on the understanding of the single-stage aging peak aging mechanism. The objective is to foster enhancements in the alloy’s performance and stimulate the advancement of the AlSi10MnMg aluminum alloy. 

This study investigates the effects of single-stage and two-stage aging processes on the properties of die-cast AlSi10MnMg aluminum alloy, examining the alloy’s microstructure, identifying the optimal aging process, and elucidating the micromechanisms responsible for enhancing the alloy’s structure and performance through aging, including precipitation and sedimentation of different phases and the role of secondary phases. Additional research scrutinizes the interplay between single-stage and two-stage aging processes and their respective impacts on the microstructure, mechanical properties, and overall performance of the die-cast AlSi10MnMg aluminum alloy. Experimental results highlighted the importance of optimizing the aging process to achieve a balance between strength, hardness, and ductility, ensuring an alloy with superior mechanical characteristics and resilience.

## 2. Experimental Procedure

**Figure 1:**
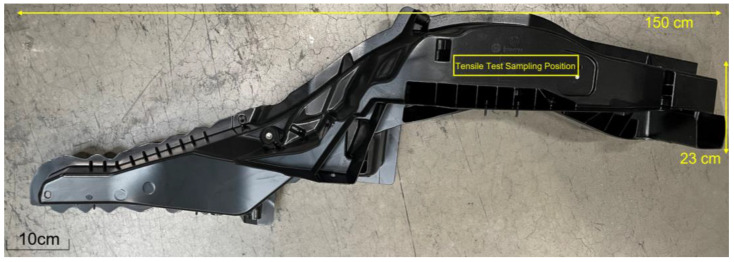
Shape specifications of longitudinal carrier and sampling position of tensile sample.

This study used optical emission spectroscopy (OES, ARL-3460, Thermo Fisher Scientific, Waltham, MA, USA) to determine the chemical composition of the AlSi10MnMg alloy, as shown in Table 1. The ingot was melted in a crucible-type resistance melting furnace at a temperature of 730 °C, stirred for approximately 10 min, and subsequently degassed with argon (Ar) for 8 min. The melt temperature was then maintained at 705 °C for insulation before high-pressure die-casting, with the mold preheated to 135 °C. Castings were produced using a horizontal cold chamber die-casting machine (DC250J-SC, Toshiba Machine Co., Ltd., Numazu, Japan). The molten metal was injected into the shot sleeve at a velocity of 0.3 m·s^−^¹, and subsequently into the die cavity at a speed of 2.5 m·s^−^¹. The test takes samples from the longitudinal carrier component from Brilliance BMW Ltd., as illustrated in Figure 1, featuring dimensions of 165 cm in length, 20 cm in width, and 3 mm in thickness. All samples underwent solution treatment at 520 °C for 2 h, followed by aging treatment at varying durations and temperatures, with individually treated specimens quenched in room-temperature water. 

**Figure 2:**
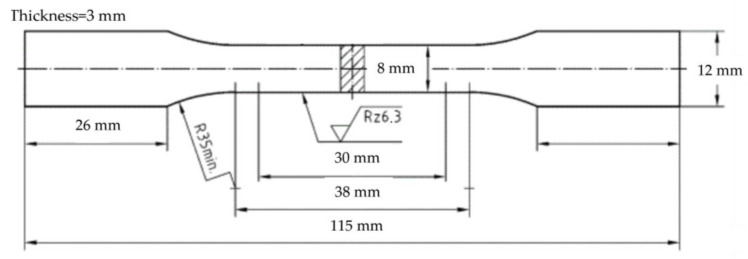
Dimensions of tensile specimen.

The tensile test specimen dimensions (DIN50125-E5, German institute for standardization, Germany) are depicted in Figure 2, and room-temperature tensile tests were performed on age-hardened samples using a universal testing machine (Z150, ZwickRoell GmbH & Co. KG, Ulm, Germany) at a strain rate of 1 mm·min^−^¹. Microstructural analysis of samples was undertaken using an optical microscope (OM, PG-2C) and scanning electron microscope (SEM, SSX-500, Shimadzu Corporation, Kyoto, Japan) after polishing and etching with 0.5% HF. Energy-dispersive spectroscopy (EDS, SSX-500, Shimadzu Corporation, Kyoto, Japan) was employed to analyze the precipitate phases within the samples post-age-hardening treatment. X-ray diffraction (XRD, D5000, Siemens AG, Munich, Germany) utilizing monochromatic Cu-Kα radiation and a step size of 0.05° was employed for phase analysis, with a scanning range of 20° to 80°. Brinell hardness measurements were performed using a microhardness tester with a 62.5 kgf load and a loading time of 30 s. Five indentations were made to provide an average hardness value during the testing process. Image pro plus software was used to measure and calculate the size and quantity of the precipitated phase. The design and range analysis of the orthogonal experiment was carried out by SPSS software (ver20, IBM Corporation, Armonk, New York, NY, USA).

## 3. Results and Discussion

### 3.1. Microstructure and Properties of Single-Aged AlSi10MnMg Alloy

**Figure 3:**
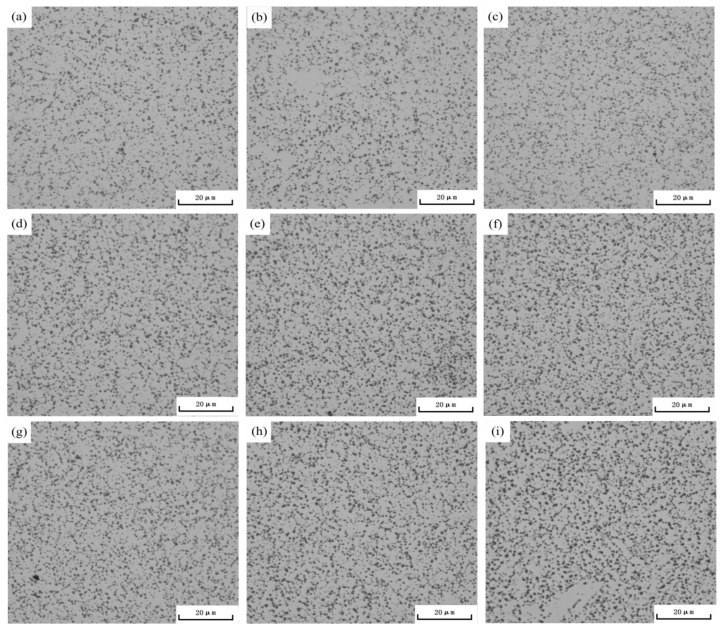
Optical micrograph of AlSi10MnMg longitudinal carrier after single-stage aging at (**a**) 165 °C × 1 h; (**b**) 165 °C × 3 h; (**c**) 165 °C × 5 h; (**d**) 180 °C × 1 h; (**e**) 180 °C × 3 h; (**f**) 180 °C × 5 h; (**g**) 195 °C × 1 h; (**h**) 195 °C × 3 h; (**i**) 195 °C × 5 h.

In the context of this study, aging temperatures of 165 °C, 180 °C, and 195 °C were chosen, with aging times of 1 h, 3 h, and 5 h. A schematic diagram of the metallographic structure is presented in Figure 3. Figure 3a–c illustrates the metallographic structures aged at 165 °C. From these images, it can be discerned that the count of secondary phase particles precipitated at grain boundaries augments with the aging time, albeit the growth rate does not show a significant rise. Figure 3d–f depicts the metallographic structures aged at 180 °C for 1 h, 3 h, and 5 h. It is evident that with increasing aging time, the secondary phase particles grow significantly from 1 h to 3 h. After 3 h of aging time, there is no substantial increase in particle quantity, and the growth rate slows down, accompanied by some degree of coarsening. Figure 3g–i displays the metallographic structures aged at 195 °C. It can be seen that when the aging time is 1 h, a certain number of secondary phase particles have already precipitated in the alloy. As time extends, the particles in the alloy’s metallographic structure do not continue to increase, but exhibit some degree of coarsening.

The obtained results shed light on aspects of the diffusion mechanism and the age-hardening process after solid solution treatment. In particular, this procedure can be compartmentalized into the dispersion of solute atoms, the inception of atomic clusters (that is, precipitate particles), and the evolution and coalescence of these particles. This process is customarily influenced by temperature and duration, accounting for the varied outcomes at different temperatures and time intervals. 

Regarding the aging treatment at 165 °C, an increase in aging time leads to a marginal increment in the number of precipitate particles at the grain boundaries, albeit the growth remains insignificant. For instance, in the transition from 1 h to 5 h of aging time, the count of precipitate particles per unit area merely elevates from 2122 PCS/mm² to 2165 PCS/mm² (shown in Table 2). This phenomenon is ascribed to the slower diffusion rate at this lower temperature, causing a delayed initiation and growth process of the precipitate phase. Consequently, even with an extended aging time, the rate of growth in the number of precipitate particles is relatively nominal. 

When the aging temperature is escalated to 180 °C, significant growth of precipitate particles is observed within the first 1 to 3 h of aging. After 3 h, the particle count does not substantially increase. The growth rate slows down, accompanied by a degree of coarsening. This trend is due to the elevated temperature accelerating the diffusion rate, thereby fostering a faster formation and growth process of the precipitate phase. Nevertheless, as the particle count increases, the driving force for diffusion diminishes, resulting in a slower growth rate. Moreover, higher temperatures encourage coalescence (coarsening) between particles. 

During the aging treatment at 195 °C, a substantial amount of precipitate particles already forms in the alloy due to the swifter diffusion rate at this increased temperature, thereby hastening the formation process. However, as time advances, the particle count in the alloy’s microstructure ceases to increase, indicating a degree of coarsening. At this temperature, the coalescence process between particles becomes more prominent, while the formation rate of new particles decelerates, leading to a halt in the growth of particle count.

In essence, these recorded outcomes emphasize the influence of diffusion mechanisms at varied temperatures and durations, alongside precipitate particle formation, growth, and coalescence processes during age-hardening. Diffusion signifies the migration of atoms or ions within the metal material. In solid metals, this migration generally transpires through vacancy mechanisms, where atoms transit to neighboring vacancies. This process is typically temperature-dependent, with elevated temperatures granting atoms increased thermal energy for faster diffusion. 

During diffusion, solute atoms might conglomerate at certain sites, forming atomic clusters, otherwise termed precipitate particles. This process unfolds over time, necessitating adequate time for solute atoms to diffuse and cluster. However, the formation and growth of particles are not unlimited processes. As the particle count escalates, the genesis of new particles becomes more challenging due to the decrease in the system’s free energy, resulting in a reduction in the driving force. Moreover, the growth rate of particles begins to decelerate as particle growth is reliant on diffusion, which inherently has velocity limitations. 

Moreover, heightened temperatures can foster particle coarsening, which pertains to the amalgamation (coalescence) of particles. Coarsening occurs as larger particles have lower surface energy than smaller particles, and the system tends to minimize its free energy, gradually disappearing smaller particles while larger particles grow. Thus, taking into consideration all these factors, the observed results can be elucidated. At the lower temperature of 165 °C, the diffusion rate is slow, giving rise to a delayed formation and growth process of the precipitate phase. The diffusion rate is expedited upon increasing the temperature to 180 °C, inducing swifter formation and growth processes. However, as the particle count inflates, the formation rate of new particles decelerates, the growth rate slackens, and particle coarseness becomes more noticeable. At the higher temperature of 195 °C, the diffusion rate is even quicker, fostering a more rapid formation process. Yet, as the particle count escalates, the formation rate decelerates, the growth rate slows down, and coarsening becomes more apparent.

**Figure 4:**
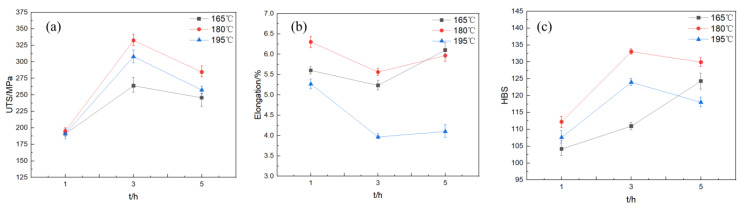
Mechanical properties of AlSi10MnMg longitudinal carrier under different aging time; (**a**) $\sigma_{UTS}$; (**b**) elongation; (**c**) hardness.

Tensile tests were conducted on the AlSi10MnMg die-cast specimens following aging. Tensile strength versus aging time curves were obtained for specimens aged at 165 °C, 180 °C, and 195 °C. Figure 4a illustrates the variations in tensile strength, elongation, and Brinell hardness values with aging time for specimens aged at 165 °C, 180 °C, and 195 °C, with holding times ranging from 1 to 5 h. As shown in the figure, with an increase in holding time, the tensile strength of specimens aged at 165 °C initially rises, reaches a peak of 263.8 MPa after 3 h of aging time, and then declines with a further increase in aging time. The minimum value of the curve is obtained at 1 h, with a tensile strength of 190.9 MPa. The tensile strength curve of specimens aged at 180 °C initially increases with a growing holding time, reaching a maximum value of 332.5 MPa at 3 h of aging time, and then decreases with a further increase in aging time. When the holding time is 5 h, the tensile strength drops to 284.6 MPa. The curve of specimens aged at 195 °C demonstrates that the tensile strength remains relatively stable with increasing aging time, with only slight fluctuations, and ultimately declines slightly at the holding time of 5 h. The maximum value of the tensile strength curve is observed at 3 h of aging time, with a value of 308.0 MPa. The minimum value is observed at the holding time of 5 h, with a tensile strength of 257.4 MPa, resulting in a varied range of approximately 50 MPa.

Examining Figure 4b, it can be seen that the overall trend of elongation at the same temperature exhibits a characteristic of first decreasing and then increasing. When the aging temperature is 165 °C, the elongation declines with increasing aging time from 1 h to 3 h. The lowest point of the elongation curve is reached at 3 h of aging time, with a value of 5.23%. Then, the elongation slightly recovers with the extension of aging time. When the aging temperature is 180 °C, the elongation curve trend demonstrates that the elongation decreases with increasing aging time from 1 h to 3 h, and the lowest point of the elongation curve is reached at 3 h of aging time, with a value of 5.56%. This value represents the minimum elongation of all single-level aged AlSi10MnMg alloy specimens. When the aging temperature is 195 °C, the elongation of the AlSi10MnMg alloy decreases with the extension of the aging time when the aging time is short. The lowest point of the elongation curve is reached at 3 h of aging time, with a value of 3.97%. Subsequently, the elongation increases with the extension of aging time and is positively correlated with aging time.

The Brinell hardness test was performed on the AlSi10MnMg alloy after aging, with hardness values selected at aging temperatures of 165 °C, 180 °C, and 195 °C. A curve of hardness versus aging time was plotted, as displayed in Figure 4c. The variation trend of Brinell hardness after aging is essentially consistent with that of tensile strength. The hardness curve initially increases and then slightly decreases with increasing aging time. The curve of 165 °C aging temperature gradually rises with the increase of holding time, reaching a maximum value of 124.3 HB at 5 h. When the aging temperature is set at 195 °C, the elongation of the AlSi10MnMg alloy diminishes with increasing aging time, especially during the shorter aging time. The maximum value appears at 3 h of holding time, with a hardness value of 123.9 HB. The curve of 180 °C aging temperature demonstrates an upward trend in hardness within 1–3 h of holding time, and then begins to decrease after 3 h. The maximum value appears at 3 h of holding time, with a Brinell hardness of 133.0 HB, and the minimum value appears at 1 h of holding time, with a Brinell hardness of 112.2 HB.

To assess the influence of aging temperature on the microstructure of the AlSi10MnMg alloy, we compared the metallographic images in Figure 3b,e,h, which correspond to aging temperatures of 165 °C, 180 °C, and 195 °C, respectively, for an aging time of 3 h. We also compared the images in Figure 3c,f,i, which correspond to aging temperatures of 165 °C, 180 °C, and 195 °C, respectively, for an aging time of 5 h. Upon comparison, it can be observed that the quantity of the secondary phase particles in the microstructure increases with increasing aging temperature at an aging time of 3 h. The maximum quantity of the secondary phase particles is observed at an aging temperature of 180 °C. As the temperature is further increased to 195 °C, the quantity of the secondary phase particles remains almost constant, but the particle size increases. At an aging time of 5 h, a large number of secondary phase particles are precipitated at 165 °C. The maximum quantity of secondary phase particles is observed at an aging temperature of 180 °C, and with a further increase in temperature, the quantity of the secondary phase particles does not increase significantly, but their size increases noticeably. 

**Figure 5:**
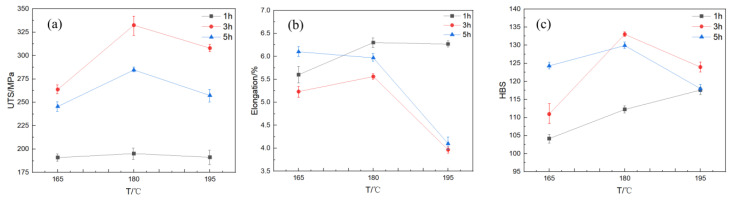
Mechanical properties of AlSi10MnMg longitudinal carrier under different aging temperatures; (**a**) $\sigma_{UTS}$; (**b**) elongation; (**c**) hardness.

Tensile tests were performed on AlSi10MnMg alloy specimens after aging at 1 h, 3 h, and 5 h. The curves of ultimate tensile strength, elongation, and Brinell hardness against aging temperature are shown in Figure 5. The results indicate that the curves of ultimate tensile strength all show a similar trend of first increasing and then decreasing, with the maximum value achieved at 180 °C, regardless of the aging time. In contrast, the elongation curves exhibit a reverse trend, first decreasing and then slightly increasing with the increase of aging temperature. The curves of elongation at 1 h and 3 h both reach the minimum value at 180 °C.

The Brinell hardness test results for the AlSi10MnMg alloy aged at different temperatures are presented in Figure 5c. The data demonstrate that aging temperature has a significant impact on the hardness of the alloy. This trend is consistent with the variation of ultimate tensile strength, indicating a close correlation between strength and hardness within the studied aging temperature range. This phenomenon can be attributed to the formation and evolution of precipitated phases during aging, as the quantity and size of the precipitated phases significantly affect the strength and hardness of the alloy. The hardness trend of the AlSi10MnMg alloy, exhibiting an initial increase followed by a decrease, is linked to the age-hardening process. Initially, as the aging period extends, solute atoms in the alloy matrix diffuse to form stable precipitates, enhancing the alloy’s hardness. However, as aging continues, these precipitates grow and coarsen, reducing the alloy’s hardness. This duality of aging effects explains the observed trend, underscoring the importance of optimal aging conditions for achieving desired alloy properties.

The formation of precipitated phases at an appropriate aging temperature can effectively improve the strength and hardness of the alloy. However, if the temperature is too high, the precipitated phases may grow excessively, leading to a decrease in both strength and hardness. Therefore, the selection of an appropriate aging temperature and holding time is critical to optimize the performance of the alloy based on specific application requirements. For example, to improve the hardness and strength of the alloy, an aging treatment at 180 °C for 3 h is recommended.

Based on the analysis of the elongation of the AlSi10MnMg alloy after aging, it was found that the elongation curve exhibits a trend opposite to that of strength and hardness, initially decreasing and then slightly increasing with the increase of aging temperature. The minimum values of elongation at 3 h and 5 h aging times are also observed at 180 °C, which corresponds to the maximum values of ultimate tensile strength and hardness. These results suggest that aging temperature significantly influences the properties of the AlSi10MnMg alloy.

**Figure 6:**
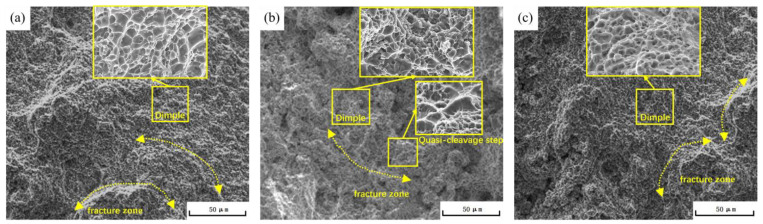
Tensile fracture surfaces SEM images of AlSi10MnMg longitudinal carrier at (**a**) 165 °C × 3 h; (**b**) 180 °C × 3 h; (**c**) 195 °C × 3 h.

The scanning electron microscopy images of the tensile fracture surfaces, shown in Figure 6, reveal a mixed fracture mode of toughness and quasi-cleavage fracture in the AlSi10MnMg alloy after aging treatment. The fracture surfaces exhibit equiaxed dimples, indicating a ductile failure mode. The number of dimples on the fracture surface at 165 °C and 195 °C aging temperatures is higher than that at 180 °C, and the dimples are deeper. This observation is consistent with the lower elongation rate of the 180 °C-aged specimen compared to that of the 165 °C-aged specimen.

According to our tensile test findings, the aging treatment considerably influences the tensile strength, elongation, and Brinell hardness of the AlSi10MnMg alloy. For samples subjected to aging treatment at temperatures of 165 °C, 180 °C, and 195 °C, the tensile strength initially escalates with increasing aging time, attains a zenith value, and subsequently begins to decline with further aging time. This trend is attributed to the precipitation of secondary phase particles in the alloy during an appropriate aging duration, thereby enhancing the alloy’s strength. Nonetheless, as the aging duration continues to extend, the secondary phase particles undergo substantial growth and coarsening, resulting in a strength decrement. The Brinell hardness trend mirrors the tensile strength since the precipitation and evolution of secondary phase particles significantly affect both the alloy’s strength and hardness. From an elongation perspective, with aging time augmentation, the elongation rate demonstrates an initial decrement followed by an increment. This is because the precipitation of secondary phase particles during the aging treatment strengthens the alloy, but concurrently reduces its ductility. With additional aging time, particle coarsening transpires, inducing a revival in the alloy’s ductility.

Regarding the microstructure, an increase in aging temperature results in an escalated number of secondary phase particles in the microstructure, attaining a maximum at 180 °C × 3 h. When the temperature is further elevated to 195 °C, the count of secondary phase particles ceases to increase, but their size does increase. This can be ascribed to the expedited diffusion of atoms at superior temperatures, facilitating more solute atoms to swiftly diffuse and precipitate as secondary phase particles, thereby promoting both particle number increment and coarsening. Scanning electron microscopy (SEM) images of the fracture surfaces depict a mixed mode of toughness and quasi-cleavage fracture in the aged AlSi10MnMg alloy. The fracture surfaces exhibit equiaxed dimples and cleavage steps, signifying a mixed fracture mode of brittleness and toughness. At 165 °C and 195 °C aging temperatures, there are more dimples on the fracture surface, appearing deeper compared to samples aged at 180 °C. This corresponds with the lower elongation observed in the sample aged at 180 °C.

In conclusion, the judicious selection of aging temperature and time is paramount for optimizing the properties of the AlSi10MnMg alloy. To increase the alloy’s hardness and strength, it is advisable to execute the aging treatment for 3 h at 180 °C.

### 3.2. Microstructure and Properties of Double-Aged AlSi10MnMg Alloy

An orthogonal experimental design employed to scrutinize the double-aging system of the AlSi10MnMg alloy facilitates the procurement of comprehensive data through fewer experimental trials, consequently resulting in time and cost efficiency. Moreover, the consideration of interaction effects between factors ensures the acquisition of more precise results. Based on the results of single-stage aging experiments, this study utilized an orthogonal experimental design method to investigate the dual-stage aging heat treatment process of the AlSi10MnMg die-casting alloy. The design incorporated four factors: first-stage aging temperature, first-stage aging time, second-stage aging temperature, and second-stage aging time, each with three levels. 

Mechanical property tests were conducted on the test specimens subjected to dual-stage aging heat treatment, and the obtained mechanical properties were analyzed to determine the optimal heat treatment conditions. Subsequently, the microstructure of the die-cast AlSi10MnMg alloy after dual-stage aging was examined. A four-factor, three-level orthogonal experimental design was implemented, resulting in a total of nine groups, as shown in Table 3. The experimental results and orthogonal analysis results are presented in Table 4. The orthogonal experimental design was based on the L9(3^4) table, with the quality value of the strength–toughness index Q serving as the evaluation criterion for mechanical properties, as demonstrated in Equation (1):

(1) $$Q=\sigma_{UTS}+k\log\left(e\right)$$

where *Q* is the strength–toughness index in MPa; $\sigma_{UTS}$ is the ultimate tensile strength, in MPa; *e* is the elongation; and *k* is a material constant, taken as 150 for the AlSi10MnMg alloy [33]. 

Range analyses were employed to assess the effects of the selected heat treatment factors on mechanical properties, and to determine the optimal aging process. The $k_{jm}$ and R values of each factor at different levels are displayed in Table 4. The range R represents the relative degree of influence on the Q quality value [34]. Furthermore, the optimal level of each factor can be ascertained by comparing $k_{jm}$, which is the sum of the indicators (the quality value of Q) of the repeated experiments corresponding to factor j and level m. The range R of each factor j (j = A, B, C, D) is calculated by Equations (2) and (3):

(2) $$\bar{k_{Jm}}=k_{jm}∕3$$

(3) $$R_{jm}=MAX\left\{\;\bar{k_{Jm}\;}\right\}-MIN\left\{\;\bar{k_{Jm}\;}\right\};\left(m=1,2,3\right)$$

where $\bar{k_{Jm}}$ is the average of $k_{jm}$ in MPa; and R is the difference between the maximum and the minimum of $\bar{k_{Jm}}$ in MPa.

Range analysis elucidates that the influence of the different factors on the Q value of the AlSi10MnMg die-casting alloy’s room-temperature tensile test is as follows: B > A > D > C. This suggests that in employing double-stage aging heat treatment for the die-cast AlSi10MnMg alloy, the degree of influence of process parameters on the alloy’s mechanical properties is in the order of first-stage aging time > first-stage aging temperature > second-stage aging time > second-stage aging temperature.

**Figure 7:**
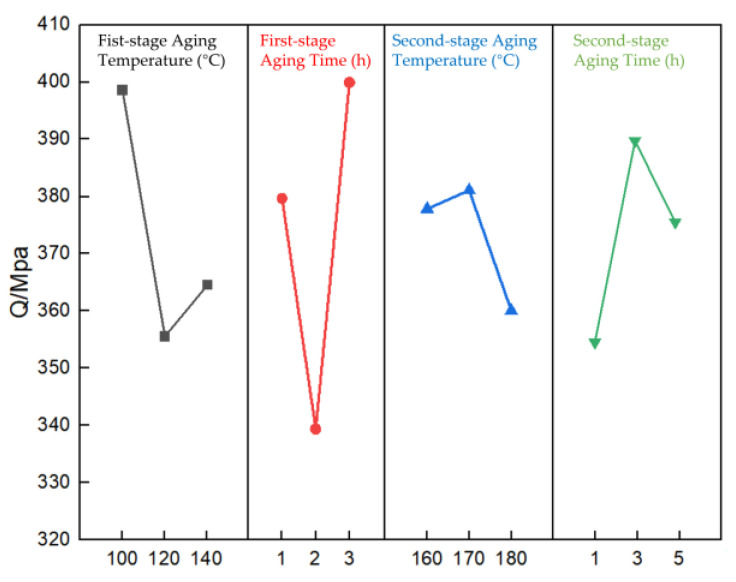
Effect of double-stage aging parameters on the Q index of die-cast AlSi10MnMg longitudinal carrier.

Figure 7 portrays the effects of first- and second-stage aging temperature and duration, along with second-stage aging temperature and period, on the Q index of AlSi10MnMg alloys. It is discernible from the figure that Q index values for the alloy, for different first-stage aging temperatures and durations, are comparatively higher than those associated with the second-stage factors.

**Figure 8:**
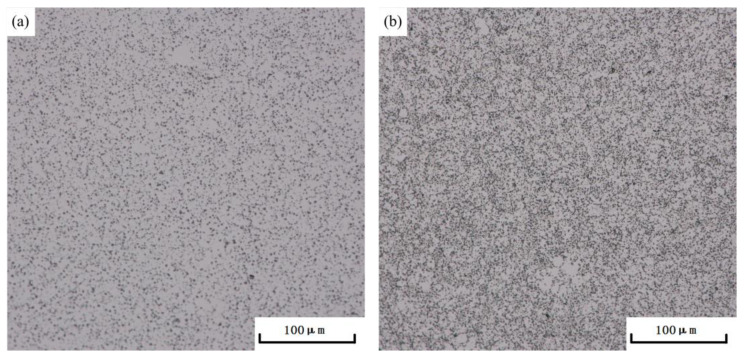
Optical micrograph of AlSi10MnMg longitudinal carrier after single-stage aging and double-stage aging at (**a**) 180 °C × 3 h and (**b**) 100 °C × 3 h + 180 °C × 3 h.

**Figure 9:**
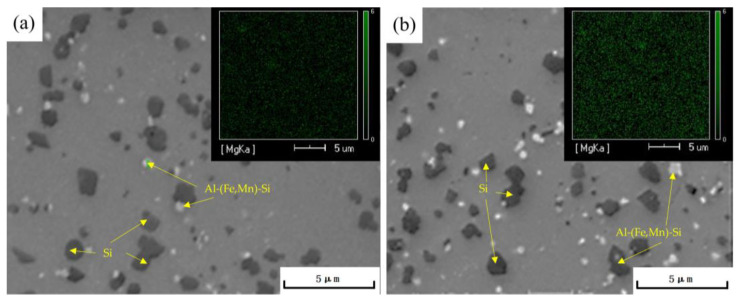
SEM images and EDS surface scanning results of AlSi10MnMg longitudinal carrier after single-stage aging and double-stage aging at (**a**) 180 °C × 3 h and (**b**) 100 °C × 3 h + 180 °C × 3 h.

Based on the range analysis of the orthogonal experiment, the double-stage aging process that achieved the optimal tensile strength for the die-cast AlSi10MnMg alloy is as follows: a first-stage aging temperature of 100 °C, a first-stage aging time of 3 h, a second-stage aging temperature of 180 °C, and a second-stage aging time of 3 h. By comparing the microstructure of the AlSi10MnMg alloy after single-stage aging (Figure 8a) and double-stage aging (Figure 8b), after double-stage aging, the precipitated phases in the alloy are more compact and uniformly distributed. The scanning electron microscopy (SEM) images and Mg element surface scanning results in Figure 9 indicate that the AlSi10MnMg longitudinal carrier, after two-stage aging treatment, has more precipitation phases that contain Mg, and the Si particles are smaller and more dispersed.

The mechanical properties of the AlSi10MnMg alloy prepared under this parameter are presented in Table 5. Through the orthogonal experiment and mechanical property tests, this study successfully determined the optimal double-stage aging heat treatment process for the AlSi10MnMg die-casting alloy. The optimal process parameters include a first-stage aging temperature of 100 °C, a first-stage aging time of 3 h, a second-stage aging temperature of 180 °C, and a second-stage aging time of 3 h. The AlSi10MnMg alloy subjected to this double-stage aging process demonstrated excellent mechanical properties. The strength–toughness index Q increases from 444.36 MPa under single-stage aging to 454.29 Mpa, with an increase of 2.3%.

### 3.3. Regulation of Precipitated Phase Particles of AlSi10MnMg Longitudinal Carrier

It is well recognized that the aging process of aluminum alloys involves sequential transformations in the microstructure: α phase spheroidal, G.P. zone acicular, β″ phase rod-like, β′ phase plate-like, equilibrium phase β [35]. The G.P. zone is an area where solute atoms are enriched and maintain a coherent relationship with the matrix; however, it has a minimal impact on the matrix’s strengthening. The β″ phase is a transitional phase that nucleates and precipitates in the G.P. zone. While maintaining complete coherence with the matrix, as the β″ phase grows, the accumulated strain and stress in the surrounding matrix increase, resulting in a strong hindrance to dislocation motion, and significantly contributing to the matrix’s strength [36]. 

With further enrichment of solute atoms, the β″ phase transforms into the β′ phase. The β′ phase maintains a partial coherent relationship with the matrix, and the elastic strain around the transitional phase reaches its maximum, thus having the strongest strengthening effect on the matrix. When the aging process is further prolonged, the β′ phase eventually transforms into the stable β phase. The β phase lacks coherence with the matrix and exerts the least strengthening effect. If the β phase coarsens during the aging process, its strengthening effect on the matrix will be further weakened, leading to overaging [37].

After aging treatment at 165 °C × 3 h, the strengthening effect of the AlSi10MnMg alloy relative to the matrix is limited and has not reached its maximum value. Under this aging process, the main strengthening mechanism in the aluminum alloy matrix is the G.P. zone. However, if the weakening effect caused by the depletion of the solid solution in the initial stage of aging is greater than the strengthening effect of precipitation in the matrix, the strength of the aluminum alloy after aging treatment will be lower than that of the untreated specimen. Increasing the aging treatment temperature and prolonging the aging treatment time significantly improves the specimen’s strength after solid solution treatment. Thus, it can be inferred that the precipitation of the β″ phase, which results in superior strength, takes place in the aluminum alloy after this aging process.

When the aging temperature is further increased, the strength of the aluminum alloy specimen continues to increase, indicating that the β″ phase precipitated in the matrix may have transformed and precipitated into the β′ phase. With the further prolongation of aging time and an increase in aging temperature, the β phase is precipitated in the matrix. After aging treatment, the strength of the aluminum alloy begins to decline. As a result, the strength value after aging treatment at 180 °C for 3–5 h and 195 °C for 1–5 h will be lower than that after aging treatment at 180 °C for 3 h.

**Figure 10:**
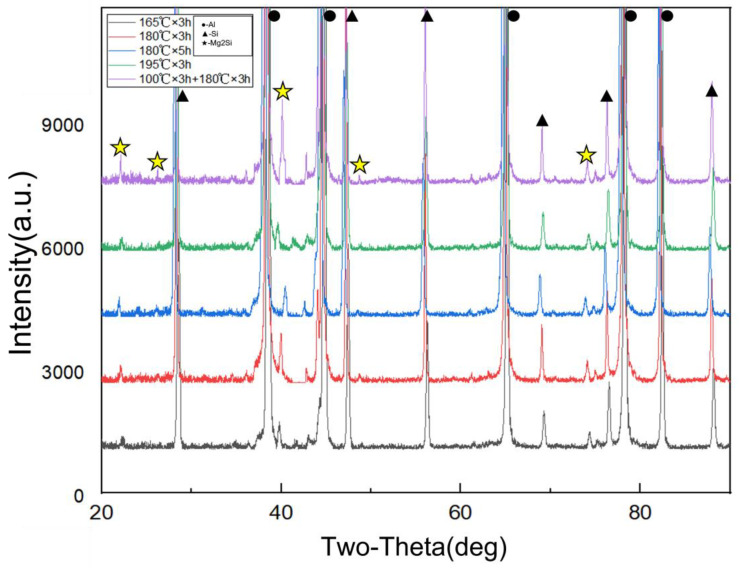
XRD test result of AlSi10MnMg longitudinal carrier.

From the XRD analysis in Figure 10, it can be observed that the Mg_2_Si phase precipitates in all of the aged AlSi10MnMg alloys, but the sharpness and area of the Mg_2_Si peaks differ. After 3 h of aging at 165 °C, the Mg_2_Si peak is relatively flat and lower, indicating that a large number of G.P. zones have precipitated, resulting in lower strength values after aging. After 3 h of aging at 195 °C × 3 h and 180 °C × 5 h, the Mg_2_Si XRD peak is the sharpest, indicating that a significant amount of stable β-Mg_2_Si has precipitated, which is considered as overaging. At 180 °C × 3 h aging, the Mg_2_Si XRD peak sharpness is moderate, which means that the Mg_2_Si in the AlSi10MnMg alloy has not completely transformed into the stable β phase, and a large amount of Mg_2_Si transitional phase exists in the alloy, resulting in higher mechanical strength values. Furthermore, the comparison of Mg_2_Si peak areas shows that at 165 °C for 3 h of aging, the Mg_2_Si peak area is 2553.15, while at 180 °C for 3 h, 180 °C for 5 h, and 195 °C for 3 h of aging, the Mg_2_Si peak area is around 3000.50, which are 2858.45, 3104.45, and 3044.60, respectively. This suggests that the content of the precipitated Mg_2_Si phase is similar under these conditions.

By utilizing a biphasic aging heat treatment, existing G.P. zones in the low-temperature primary aging region do not dissolve, but grow slowly and stabilize. Meanwhile, the remaining supersaturated solid solution continues to decompose, precipitating G.P. zones and a small amount of metastable phase. Mg and other atoms in the alloy continue to precipitate from the solid solution and diffuse into G.P. zones and metastable phases, thereby enhancing their stability. Therefore, in the subsequent high-temperature secondary aging process, the coarsening rate of precipitated phases within the grain decelerates, permitting longer aging times under equivalent strength conditions. However, the precipitated phases along grain boundaries become larger, and the spacing between particles widens with longer aging times. Consequently, after the biphasic aging treatment, the alloy’s elongation significantly improves, which is consistent with the experimental XRD results.

## 4. Conclusions

Following the analysis of tensile and hardness data, metallographic images, and scanning electron microscopy (SEM) images of the AlSi10MnMg longitudinal carrier under varying aging processes, we can derive several conclusions:

1. Under single-stage aging treatment, the AlSi10MnMg longitudinal carrier exhibits the highest strength when aged at 180 °C × 3 h, with a tensile strength of 332.5 MPa, a macroscopic Brinell hardness of 133.0 HB, and an elongation of 5.56%.
2. With an increase in aging time, the tensile strength and hardness of the AlSi10MnMg longitudinal carrier display an increasing trend followed by a decreasing trend, while the elongation rate shows a decreasing trend followed by an increasing trend. When the aging time is constant, as the solution treatment temperature increases, both the strength and hardness demonstrate an increasing trend followed by a decreasing trend.
3. Through metallographic observation, the amount of secondary phase particles gradually increases and diffuses at grain boundaries with an increase in aging temperature and holding time. The secondary phase particle precipitation rate slows down as aging continues, and the quantity reaches stability. As the aging temperature and time increase, the secondary phase particles begin to grow, and the strengthening effect on the alloy begins to weaken.
4. The fracture surface morphology of the tensile samples was observed using scanning electron microscopy, and the fracture surface exhibited mixed fracture characteristics, with the presence of ductile dimples and brittle cleavage steps.
5. Range analysis was used to analyze the tensile strength results after double-stage aging, and the degree of influence of different parameters on the mechanical properties of the alloy follows this order: the first-stage aging > the temperature of the first-stage aging > the time of the second-stage aging > the temperature of the second-stage aging.
6. The optimal double-stage aging process for obtaining the highest tensile strength of the AlSi10MnMg longitudinal carrier is as follows: the temperature of the first-stage aging is 100 °C, the time of the first-stage aging is 3 h, the temperature of the second-stage aging is 180 °C, and the time of the second-stage aging is 3 h. Compared to single-stage aging, the strength–toughness index increased by 2.3%.

## Figures and Tables

**Table 1:** Chemical composition of the AlSi10MnMg longitudinal carrier (wt. %).

Si	Mg	Mn	Fe	Mn/Fe	Ti	Sr	Zn	Cu	Al
10.5	0.26	0.52	0.20	2.60	0.04	0.017	0.000930	0.00189	88.4

**Table 2:** Precipitated phase size and quantity of die-cast AlSi10MnMg longitudinal carrier.

Aging Process	Mean Diameter of Precipitated Phase (μm)	Average Area of Precipitated Phase (μm^2^)	Number of Precipitated Phases per Unit Area (PCS/mm^2^)
165 °C × 1 h	1.614	5.016	2122
165 °C × 3 h	1.664	5.102	2113
165 °C × 5 h	1.697	5.121	2165
180 °C × 1 h	1.694	5.992	2278
180 °C × 3 h	1.693	6.074	2395
180 °C × 5 h	1.843	6.731	2399
195 °C × 1 h	1.708	6.118	2221
195 °C × 3 h	1.767	6.383	2279
195 °C × 5 h	1.882	6.742	2297

**Table 3:** Orthogonal table for bipolar aging of die-cast AlSi10MnMg longitudinal carrier.

Factor	A Fist-Stage Aging Temperature (°C)	B First-Stage Aging Time (h)	C Second-Stage Aging Temperature (°C)	D Second-Stage Aging Time (h)
1	100	1	165	1
2	120	2	180	3
3	140	3	195	5

**Table 4:** Orthogonal test results of Q index of die-cast AlSi10MnMg longitudinal carrier.

Factor	A	B	C	D	Q/MPa	$\sigma_{UTS}/\mathrm{MPa}$	Elongation/%
A1B1C1D1	100	1	160	1	390.28	265.4	6.8
A1B2C2D2	100	2	170	3	390.01	290.6	4.6
A1B3C3D3	100	3	180	5	414.69	321.2	4.2
A2B1C2D3	120	1	170	5	373.03	269.5	4.9
A2B2C3D1	120	2	180	1	289.80	169.9	6.3
A2B3C1D2	120	3	160	3	404.86	269.4	8.0
A3B1C3D2	140	1	180	3	375.72	259.0	6.0
A3B2C1D3	140	2	160	5	338.40	231.0	5.2
A3B3C2D1	140	3	170	1	380.22	285.2	4.3
K1	1194.98	1139.03	1133.54	1060.30	-	-	-
K2	1067.69	1018.21	1143.26	1170.59	-	-	-
K3	1094.34	1199.77	1080.21	1126.12	-	-	-
k1	398.33	379.68	377.85	353.43	-	-	-
k2	355.90	339.40	381.09	390.20	-	-	-
k3	364.78	399.92	360.07	375.37	-	-	-
R	42.43	60.52	21.02	36.76	-	-	-
Correlation degree	B > A > D > C						
Optimal combination	A1B3C2D2						

**Table 5:** Tensile test results of die-cast AlSi10MnMg longitudinal carrier under the optimal combination.

$\sigma_{UTS}/\mathrm{MPa}$	HB	Elongation/%
326.60	128.4	7.1

## Data Availability

Not applicable.
